# ChatGPT as an AI-Enabled Educational Resource in Nursing Practice: Scoping Review of Uses, Outcomes, and Implementation Challenges

**DOI:** 10.2196/79551

**Published:** 2026-03-09

**Authors:** Selviana Anwar, Saldy Yusuf, Maria Kurnyata Rante Kada, Farawansah Mustafa

**Affiliations:** 1 Faculty of Nursing, Hasanuddin University, Jl Perintis Kemerdekaan, KM 10, Makassar, Indonesia, 62-81241841800; 2 Wahidin Sudirohusodo General Hospital, Makassar, Indonesia; 3 Indonesian Diabetic Foot Care Research Group, Makassar, Indonesia; 4 STIKES Panakukkang, Makassar, Indonesia

**Keywords:** artificial intelligence, Chat GPT, nursing education, patient education, nurse, scoping review

## Abstract

**Background:**

High-quality nursing services are essential for improving patient satisfaction and health outcomes. Today, artificial intelligence (AI) applications such as ChatGPT offer potential solutions to enhance patient education and assist nurses in providing more accurate and personalized information. Despite its promising potential in nursing education, concerns regarding information accuracy, privacy, and ethical considerations must be addressed.

**Objective:**

This scoping review aimed to map the current evidence on the use of ChatGPT (OpenAI) as an educational resource in nursing practice, focusing on its educational functions, reported outcomes, and implementation challenges.

**Methods:**

The literature search was conducted using 3 databases (PubMed, Scopus, and ProQuest). Following the Population, Concept, and Context framework. Inclusion criteria encompassed studies published between 2019 and 2025, in English, and available in full text. The PRISMA-ScR (Preferred Reporting Items for Systematic reviews and Meta-Analyses extension for Scoping Reviews) guideline was used to guide the screening and selection process.

**Results:**

We included 20 articles and synthesized four main findings: (1) AI in patient education and simplification of medical information, (2) AI in clinical decision-making and patient monitoring, (3) AI in nursing education, and (4) challenges and prospects of AI in nursing. Across studies, commonly reported limitations involved response accuracy inconsistencies, ethical concerns, and the absence of standardized implementation guidelines.

**Conclusions:**

ChatGPT shows promise as an adjunct educational resource in nursing practice, particularly for information accessibility and learner engagement. Nevertheless, its use requires professional oversight, ethical safeguards, and further implementation-focused research.

## Introduction

The quality of nursing care plays a central role in patient satisfaction and holistic health outcomes. However, persistent challenges, including workforce shortages, increasing workloads, and the growing burden of chronic disease continue to constrain the delivery of effective education in clinical settings [1]. In addition, internet-based nursing care models face implementation barriers related to unclear policies and limited practical guidance, further limiting their effectiveness [2]. Together, these pressures underscore the need for scalable and responsive educational approaches that are feasible within time-constrained nursing environments.

According to existing studies, nursing education in hospitals has been shown to impact patient health outcomes significantly [3]. Virtual education modalities, including teleconferencing and web-based platforms, have expanded access to nursing education and professional development [3]. However, both face-to-face and teleconference-based approaches remain limited by time constraints, logistical challenges, and reduced opportunities for hands-on skill development in clinical settings [4,5]. Despite expanding access, these technology-mediated educational approaches remain largely static and dependent on scheduled interactions, highlighting persistent gaps in timely, adaptive, and individualized educational support within nursing practice.

Recent advances in artificial intelligence (AI), particularly large language models, have introduced new possibilities for addressing these gaps. The need to develop a more structured and systematic approach to nursing education has drawn attention to AI as a potentially more adaptive and interactive educational tool that can complement existing technologies [6]. With the advancement of IT, AI has been explored for its capacity to automate selected administrative processes, support information delivery, and assist educational activities, thereby potentially alleviating educational bottlenecks within constrained nursing workflows [7,8]. Within this context, ChatGPT is primarily being explored not as a solution to workforce shortages per se, but as a supplementary educational resource that may support nurses and patients by enabling on-demand access to information and educational clarification when direct professional interaction is limited. ChatGPT has attracted growing interest for its potential to support personalized learning experiences, accelerating feedback delivery, and enhancing engagement in educational settings [9]. However, these applications remain largely exploratory and are accompanied by concerns regarding response accuracy, ethical accountability, data privacy, and contextual appropriateness in clinical education.

Current trends indicate that the use of AI, particularly ChatGPT, in nursing education and practice is increasing. Nevertheless, significant challenges remain, including ethical issues related to privacy, the risk of plagiarism, and the potential for generating inaccurate or misleading information [10]. Furthermore, although many health care professionals (HCPs) express interest in integrating ChatGPT into their practice, uncertainty persists regarding its long-term impact and appropriate role within nursing education and clinical workflows [11].

Given these gaps, a comprehensive synthesis of current evidence is required to clarify how ChatGPT is being used as an educational resource in nursing practice, what educational outcomes have been reported, and what challenges accompany its implementation [10]. A scoping review is particularly appropriate for this purpose, as it allows for mapping emerging evidence, identifying patterns and knowledge gaps, and summarizing a rapidly evolving body of literature characterized by methodological heterogeneity.

Although interest in ChatGPT within nursing has increased rapidly since 2023, existing studies remain fragmented, often focusing on conceptual perspectives or isolated applications rather than providing an integrated overview [9]. To date, no scoping review has systematically mapped how ChatGPT is used as an educational resource in nursing practice, what outcomes have been reported, and what implementation challenges persist. This scoping review aimed to map the current evidence on the use of ChatGPT as an educational resource in nursing practice, focusing on its educational functions, reported outcomes, and implementation challenges.

## Methods

### Overview

We adopt the framework by Arksey and O’Malley, which includes 5 key stages in the implementation of a scoping review [12]. This framework was selected to map the existing literature, identify research gaps, and provide an overview of the available evidence related to the research objectives. The methodological structure used consists of 5 stages: stage 1—defining the research question; stage 2—identifying studies relevant to the research question; stage 3—selecting studies to be included in the review; stage 4—mapping data from the included studies; and stage 5—synthesizing, summarizing, and reporting the findings.

### Stage 1: Research Question

The research question for this scoping review is: how has ChatGPT been used as an educational resource in nursing practice, and what educational outcomes and implementation challenges have been reported?

### Stage 2: Relevant Studies and Search Strategy

A systematic search was conducted across 3 electronic databases: PubMed, Scopus, and ProQuest. The search was performed on (January 19, 2025) to identify relevant literature published in the last 6 years.

The inclusion and exclusion criteria for this review were established according to the Population, Concept, Context model. The search strategy was built using the Population, Concept, Context framework with specific keywords and Boolean operators as lists (Textbox 1).

Textbox 1. **Search strategies used for each database.**

**Search strategy**

- PubMed: (((Health Education”[Mesh]) OR “Health Promotion”[Mesh]) OR (education[Title] OR counseling[Title] OR promotion[Title])) AND ((artificial intelligence [MeSH Terms]) OR (ChatGPT[Title])) AND (nursing[Title] OR nurse[Title])
- Scopus: (health education OR health promotion OR education OR counseling OR promotion AND artificial intelligence OR ChatGPT AND nursing OR nurse)
- ProQuest: abstract(Health Education OR Health Promotion OR education OR counseling OR promotion) AND abstract (artificial intelligence OR ChatGPT) AND abstract(nursing OR nurse)

The search was limited to studies published between 2019 and 2025, written in English, and with full-text availability.

### Stage 3: Study Selection

#### Study Extraction From Databases

**Figure 1.**
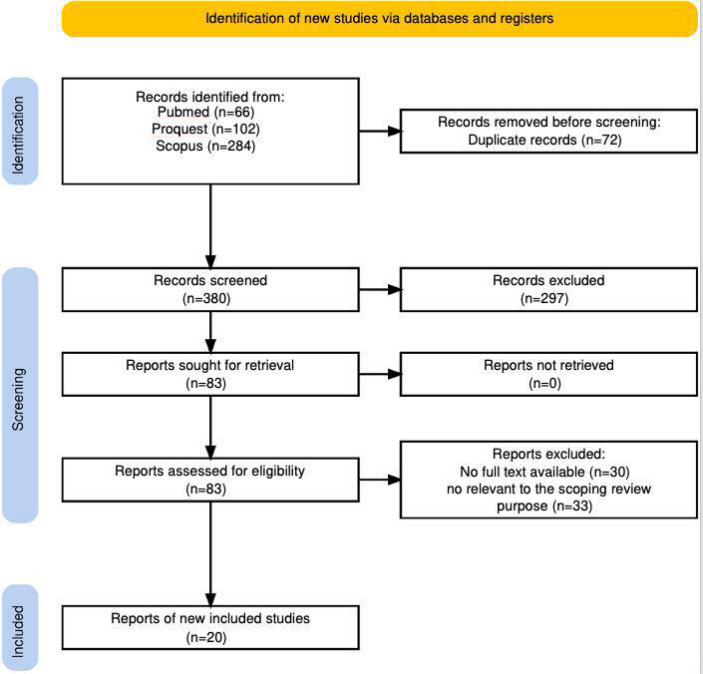
PRISMA (Preferred Reporting Items for Systematic Reviews and Meta-Analyses) flow diagram.

The study selection process rigorously followed the PRISMA-ScR (Preferred Reporting Items for Systematic Reviews and Meta-Analyses extension for Scoping Reviews) guidelines [12]. All stages of identification, screening, eligibility assessment, and inclusion were documented and reported using a PRISMA-ScR flow diagram (Figure 1). The detailed PRISMA-ScR checklist is presented in Multimedia Appendix 1.

After the initial search, all records were imported into Rayyan for deduplication and screening. Duplicates were removed automatically and verified manually. Titles and abstract screening was conducted independently by 2 reviewers (FF and MK). Following a pilot screening of 50 randomly selected articles to ensure shared understanding of eligibility criteria. To minimize selection bias, the blinding function in Rayyan was applied where feasible. Full texts of potentially eligible studies were then assessed independently.

Disagreements at the title or abstract or full-text screening stages were resolved through discussion and consensus. When consensus could not be reached, a third reviewer (SA) acted as an arbitrator [13]. Two reviewers (FF and MK) independently extracted data, and discrepancies were resolved through consensus with a third reviewer (SA). Although formal interrater reliability statistics were not calculated, this approach aligns with established scoping review methodology.

### Stage 4: Data Mapping

Data from the included studies were systematically extracted into a standardized data-charting form developed by the team. The following variables were extracted: (1) authors and publication year, (2) country of origin, (3) study design, (4) study population and sample size, (5) specific AI technology or intervention investigation (ChatGPT), (6) form of educational resource, and (7) key findings relevant to the review objectives. The data charting form was piloted on 5 included studies and refined accordingly [6,8,14-16]. The article synthesis process was supported by an AI-based tool (Elicit.ai), which was used to assist with organizing and summarizing study findings, while the final interpretation and thematic grouping were conducted by the researchers. Data synthesis was conducted using a descriptive and iterative thematic grouping approach rather than formal qualitative coding, in accordance with the exploratory purpose of scoping reviews

The synthesized data are presented in Table 1.

**Table 1.** Description of study designs

Number	Authors and year of publication	Number of study	Study design
1	O’Connor et al (2024) [17], Lora and Fo (2024) [18], Zhou et al (2024) [19], Lukkahatai and Han (2023) [14], Buchanan et al (2021) [15], Nashwan et al (2024) [20], Martinez-Ortigosa et al (2023) [8], and Groeneveld et al (2024) [21]	8	Perspective reviews or review papers
2	Huang and Lee (2024) [22] and Chang et al (2024) [6]	2	Quasi-experimental design
3	Issa et al (2024) [23] and Sezgin et al (2025) [24]	2	Cross-sectional survey
4	Alanzi (2023) [25] and Rony et al (2024) [26]	2	Qualitative study
5	Chen et al (2023) [16] and Seo and Kim (2024) [27]	2	Bibliometric analysis and topic modeling analysis
6	Yüceler Kaçmaz et al (2024) [28]	1	Methodological study
7	Zeng et al (2024) [29]	1	A comparative study
8	Moons and Van Bulck 2024 [30] Shorey et al 2019 [31]	2	Development studies

### Stage 5: Thematic Summary and Key Findings

The findings from the literature were used to identify outcomes based on emerging keywords. This review will analyze all articles through titles, abstracts, and full texts, followed by a check to identify any differences and duplicates. All articles analyzed contain information regarding AI technology as a source of nursing education.

### Ethical Considerations

This study was exempted from the evaluation requirements by the Institutional Review Board for Human Subjects because the articles analyzed only disclosed anonymized information or did not contain personal data that could identify specific individuals.

## Results

### Study Characteristics

This scoping review included 20 studies [6,8,14-31] published between 2019 and 2025, conducted across diverse geographic regions (Table 2). The predominance of studies from Asia reflects varying levels of AI adoption and research maturity rather than differences in effectiveness across health care systems. A detailed breakdown of the geographic distribution of the included studies is provided (Table 2).

**Table 2.** Geographic distribution of included studies (n=20).

Country	Number of study	Author (year)
Multi-country studies	6	Lukkahatai and Han (2023) [14], Nashwan et al (2024) [20], Lora and Fo (2024) [18], O’Connor et al (2024) [17], and Sezgin et al (2025) [24], Issa et al (2024) [23]
Asia studies		
China	3	Chen et al (2023) [16], Zeng et al (2024) [29], and Zhou et al (2024) [19]
Turkey	1	Yüceler Kaçmaz et al (2024) [28]
Taiwan	2	Chang et al (2024) [6] and Huang and Lee (2024) [22]
Bangladesh	1	Rony et al (2024) [26]
Saudi Arabia	1	Alanzi (2023) [25]
Singapore	1	Shorey et al (2019) [31]
South Korea	1	Seo and Kim (2024) [27]
Europe and North America studies		
Spain	1	Martinez-Ortigoza et al (2023) [8]
Belgium	1	Moons and Van Bulck (2024) [30]
Netherlands	1	Groeneveld et al (2024) [21]
Canada	1	Buchanan et al (2021) [15]

### Study Design

Overall, 20 articles were analyzed, and various research designs were used to evaluate the application of AI in health care. Findings were interpreted narratively by study design to provide a clearer understanding of the strength and nature of the available evidence (Table 2).

### Use of AI Technology

Across the included studies, AI was applied in nursing practice and education primarily as a supportive educational and informational tool, rather than as an autonomous clinical decision-maker [17,19]. The AI technologies identified can be broadly categorized into three functional groups: (1) large language model–based systems, (2) machine learning (ML)–driven analytic tools, and (3) interactive educational technologies [8,16,27].

First, large language models, particularly ChatGPT (GPT-3.5 and GPT-4), were the most frequently reported AI tools [6,19,29,30]. These systems were used to provide on-demand explanations, support information-seeking behaviors, and assist with educational clarification for nurses, patients, and caregivers [24,25,32]. Studies consistently described ChatGPT as facilitating access to health information, supporting learning activities, and assisting communication in both educational and clinical contexts[6,24,29].

In parallel, several studies reported the use of ML, natural language processing (NLP), and robotic process automation to support clinical monitoring, risk prediction, and information management [8,17,20]. These applications focused on enhancing early detection, optimizing workflow efficiency, and supporting decision-making processes by synthesizing large volumes of clinical data [21,26]. Importantly, these technologies were described as augmenting, rather than replacing, professional judgment [17,21].

A third group of applications involved interactive educational technologies, including AI-based chatbots, virtual avatars, educational robots, AI-generated e-books, and virtual or augmented reality tools [16,22,31]. These technologies were primarily used in nursing education to support collaborative learning, simulation-based training, and communication skill development, particularly in pediatric and remote learning contexts [16,22,31].

Overall, the reviewed studies indicate that AI technologies are implemented heterogeneously across nursing contexts, with their role largely confined to information support, educational facilitation, and workflow assistance [7,19,32]. Direct clinical decision-making autonomy was not reported, and professional oversight remained a consistent requirement across applications [17,21,25].

### Patient Education Outcome

Across the included studies, reported outcomes related to patient education varied considerably in scope and methodological rigor. Educational outcomes associated with AI-supported patient education were reported across cognitive, behavioral, and informational domains, although the depth and rigor of outcome measurement varied substantially between studies [7,19]. Consequently, outcome evidence was interpreted cautiously and weighted according to study design (Table 3).

**Table 3.** Patient education outcomes of artificial intelligence-based interventions in nursing practice and education (N=20).

No	Author (year)	AI^a^ tool	Educational outcome domain	Key outcomes (reported)
1	Lukkahatai and Han (2023) [14]	AI chatbots and virtual assistants	Patient education and safety	AI-supported education tools were associated with improved access to patient education and reduced safety risks, including a pressure injury prediction recall of 87.2%, as reported descriptively.
2	Chang et al (2024) [6]	ChatGPT	Cognitive and learning outcomes	The intervention group showed higher critical thinking (mean 4.73, SD 0.44), problem-solving ability (mean 4.53, SD 0.71), and learning satisfaction (mean 4.69, SD 0.46) compared with controls (*P*<.001).
3	Martinez-Ortigosa et al (2023) [8]	ML^b^, NLP^c^, and RPA^d^	Education and clinical decision support	AI-based educational and decision-support systems were associated with reported improvements in diagnostic accuracy of approximately 12% across included studies.
4	Chen et al (2023) [16]	AI robots and chatbots	Educational innovation trends	Publication trends increased from 26 articles in 2001–2010 to 49 articles in 2018–2021, indicating growing research attention to AI-assisted education.
5	Buchanan et al (2021) [15]	VR/AR^e^, avatars, and AI systems	Curriculum and learning support	The review identified potential educational benefits of AI in nursing curricula, without reporting quantitative outcome measures.
6	Zeng et al (2024) [29]	GPT-3.5 and GPT-4	Information quality	GPT-4 responses were rated higher in quality and accuracy than GPT-3.5 responses by neurologists, with statistically significant differences (*P*<.001).
7	Moons and Van Bulck (2024) [30]	ChatGPT and Google Bard	Readability and comprehension	ChatGPT reduced text readability from grade 11 to grade 9, while Google Bard achieved a grade 6 readability level with a text length reduction of 83%.
8	Rony et al (2024) [26]	AI systems	Educational and decision support	Most participants reported positive perceptions of AI for educational support, while also expressing concerns regarding about reduced human interaction (*P*<.05).
9	Nashwan et al (2024) [20]	AI systems	Education and workflow support	AI systems were described as supporting educational workflows and documentation efficiency, with outcomes discussed conceptually rather than quantitatively.
10	Alanzi (2023) [25]	ChatGPT	Educational support and engagement	Health care professionals reported improved efficiency and engagement in patient education during teleconsultation, alongside concerns about diagnostic reliability.
11	Groeneveld et al (2024) [21]	AI monitoring	Patient education and self-management	AI-based monitoring tools were accepted as supportive educational aids in long-term care, but not as replacements for direct nurse-patient interaction.
12	Lora and Foran (2024) [18]	AI analytics	Education and anxiety reduction	The reviewed studies suggested reductions inpatient anxiety and improved information delivery, although however, reported outcomes were heterogeneous, with one study demonstrating a significant reduction in preoperative anxiety through via an AI chatbot (P<.001).
13	O’Connor et al (2024) [17]	ML and NLP	Patient education and prediction	AI applications improved predictive accuracy and educational support in cancer nursing, primarily based on secondary evidence synthesis.
14	Shorey et al (2019) [31]	AI virtual patient	Communication education	Nursing students reported higher communication confidence and self-efficacy following use of an AI-based virtual counseling application.
15	Zhou et al (2024) [19]	ChatGPT	Educational use	Sixty-seven percent of the included studies focused on educational applications of ChatGPT, with outcomes mainly primarily reported as perceived benefits.
16	Seo and Kim (2024) [27]	Generative AI	Educational trends	Topic modeling identified patient education and simulation-based learning as dominant research themes, without direct outcome evaluation.
17	Sezgin et al (2025) [24]	ChatGPT, Bard, and others	Information quality	ChatGPT achieved higher scores for accuracy (mean 2.71, SD 0.235), clarity (mean 2.73, SD 0.271), completeness (mean 0.815, SD 0.203), and clinical use (mean 3.81, SD 0.544) compared with other models.
18	Issa et al (2024) [23]	Conceptual survey on AI literacy and attitudes	Knowledge Attitude Perceived barriers	Low AI literacy (66.4%), positive attitude (51.2%), support AI integration (77.6%), main barriers: lack of training and awareness.
19	Huang and Lee (2024) [22]	AI eBook	Communication and anxiety	The AI-based intervention was associated with reduced children’s fear responses (β = −1.540, P<.05) and improved nursing students’ communication self-efficacy.
20	Yüceler Kaçmaz et al (2024) [28]	ChatGPT-assisted material	Understandability and actionability	AI-assisted education materials demonstrated high understandability (81.91%) and actionability (85.33%) based on PEMAT^f^ scores.

a AI: artificial intelligence.

b ML: machine learning.

c NLP: natural language processing.

d RPA: robotic process automation.

e VR/AR: virtual or augmented reality devices.

f PEMAT: Patient Education Materials Assessment Tool.

### Cognitive and Learning-Related Outcomes

Quantitative evidence of cognitive outcomes was primarily reported in quasi-experimental studies. A controlled study in Taiwan demonstrated that integrating ChatGPT into nursing education significantly improved critical thinking (mean 4.73, SD 0.44), problem-solving skills (mean 4.53, SD 0.71), and learning satisfaction (mean 4.69, SD 0.46) compared with a control group (*P*<.001) [6]. These findings suggest that large language models can enhance higher-order learning processes when embedded within structured educational designs. Trend and bibliometric analyses further indicated a rapid increase in the application of AI-based educational tools, including chatbots and educational robots, within nursing and Science, Technology, Engineering, and Mathematics education since 2018 [16]. While these analyses do not measure learning outcomes directly, they reflect growing institutional and academic interest in AI-supported education.

### Readability, Comprehension, and Information Accessibility

Several studies evaluated informational outcomes by assessing improvements in the readability and comprehensibility of patient education materials. ChatGPT was shown to reduce the reading level of medical texts from grade 11 to grade 9 while largely preserving content integrity, whereas Google Bard achieved lower reading levels at the expense of substantial content reduction [30]. These findings highlight the potential of AI to improve accessibility of patient education materials, while also underscoring variability across AI tools. Similarly, an AI-assisted educational intervention for ostomy patients demonstrated high understandability (mean 81.91%, SD 19.05%) and actionability (mean 85.33%, SD 26.44%) scores using the Patient Education Materials Assessment Tool (Agency for Healthcare Research and Quality) [28]. These outcomes suggest that AI may support tailored patient education in contexts requiring structured and repetitive informational support.

### Clinical and Caregiver-Oriented Educational Outcomes

In clinical and caregiving settings, AI-supported education was associated with improved perceived quality, clarity, and usefulness of health information. Comparative evaluations of large language model–based tools found that ChatGPT outperformed alternative platforms in accuracy, completeness, and clinical use when responding to caregiver information needs, particularly in pediatric oncology contexts [24]. However, these studies emphasized that AI-generated information should be interpreted as supportive, rather than definitive, educational guidance. Clinical education–related outcomes were reported in disease-specific contexts. In Alzheimer disease management, GPT-4 generated responses rated by neurologists as significantly higher in quality than those produced by GPT-3.5 (*P*<.001), suggesting potential value in supporting caregiver education for complex chronic conditions [29]. Nonetheless, these findings were based on controlled evaluations and did not assess downstream clinical or behavioral outcomes.

### Psychosocial and Engagement-Related Outcomes

Behavioral and psychosocial outcomes were primarily reported in qualitative and development studies. AI-generated educational e-books significantly reduced fear-related behavioral responses among pediatric patients undergoing medical procedures (β=−1.540; *P* <.05), while also enhancing nursing students’ self-efficacy in therapeutic communication [22]. In teleconsultation settings, HCPs reported improved engagement and efficiency in patient education, although concerns regarding diagnostic accuracy and contextual appropriateness persisted [25]. Overall, patient education outcomes associated with AI use were most consistently demonstrated in controlled educational settings and condition-specific interventions. However, the predominance of descriptive and short-term studies limits conclusions regarding sustained clinical impact. Collectively, the evidence suggests that AI-supported patient education may contribute to improved psychosocial and behavioral outcomes in specific contexts, although evidence of sustained impact remains limited.

### Key Findings

**Figure 2.**
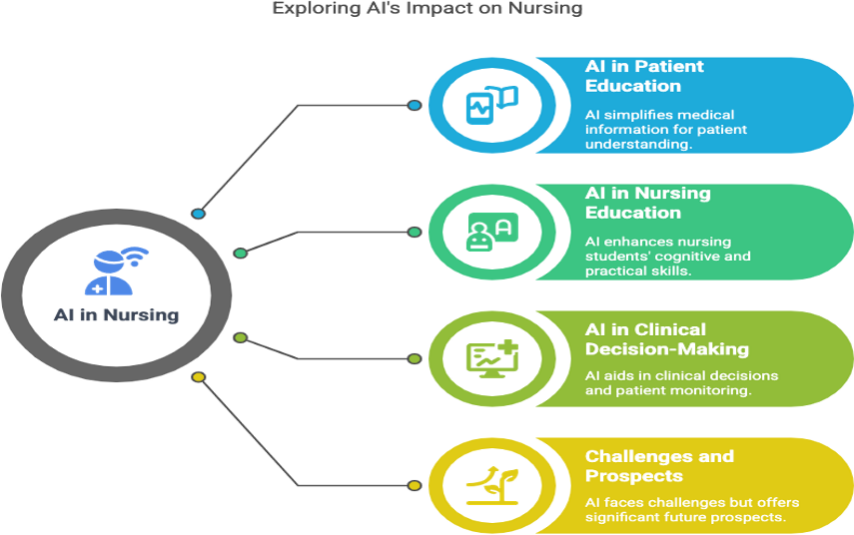
Four main areas that impact artificial intelligence in various aspects of nursing. AI: artificial intelligence.

This scoping review highlights various implementations of AI technology in nursing education and clinical practice, reviewing 20 studies that assess the impact of AI in multiple aspects of nursing. The key findings from this review can be categorized into four main areas; (1) AI in patient education and simplification of medical information [17,23,24,28,30,31], (2) AI in clinical decision-making and patient monitoring [8,20,29], (3) AI in nursing education [6,15,16,18,19,22,27] and (4) challenges and prospects of AI in nursing [14,21,25,26](Figure 2).

To enhance clarity and readability, the main findings of this review are summarized in Table 4, highlighting 4 key areas of AI application in nursing along with the associated evidence.

**Table 4.** Summary of key findings on the use of artificial intelligence in nursing education and clinical practice.

Main area	Included studies	Key findings
AI^a^ in patient education and simplification of medical information	Moons and Van Bulck (2024) [30], Yüceler Kaçmaz et al (2024) [28], Shorey et al (2019) [31], Issa et al (2024) [23], Sezgin et al (2025) [24], and O’Connor et al (2024) [17]	AI tools, including large language models, were primarily used to simplify medical information and adapt language complexity to patients’ educational levels. Studies reported improved comprehensibility of patient education materials and enhanced patient understanding of care, particularly in chronic and postsurgical contexts such as ostomy care.
AI in clinical decision-making and patient monitoring	Martinez-Ortigosa et al (2023) [8], Zeng et al (2024) [29], and Nashwan et al (2024) [20]	AI applications supported early disease detection, diagnostic accuracy, and clinical decision-making through machine learning and natural language processing. Evidence indicated that AI-assisted systems could provide timely clinical insights and function as supportive tools in patient monitoring and disease management, including neurological conditions.
AI in nursing education	Chang et al (2024) [6], Zhou et al (2024) [19], Huang and Lee (2024) [22], Chen et al (2023) [16], Seo and Kim (2024) [27], Lora and Foran (2024) [18], and Buchanan et al (2021) [15]	AI-based educational tools, such as chatbots, virtual patients, and AI-driven learning platforms, were associated with improvements in nursing students’ critical thinking, problem-solving, communication skills, self-efficacy, and learning satisfaction. Research trends demonstrated a marked increase in AI integration in nursing education since 2018, particularly in technology-enhanced and remote learning environments.
Challenges and prospects of AI in nursing	Rony et al (2024) [26], Alanzi (2023) [25], Groeneveld et al (2024) [21], Lukkahatai and Han (2023) [14]	Key challenges included ethical concerns, data privacy, information accuracy, and limitations in providing personalized and empathetic care. While AI showed promise in supporting education and decision-making, studies highlighted the need for cautious implementation to avoid reduced human interaction and potential dissemination of inaccurate or context-inappropriate information.

a AI: artificial intelligence.

### The Role of AI in Patient Education and Simplification of Medical Information

AI has been used to simplify medical information to make it more understandable for patients. Studies show that ChatGPT and Google Bard can adjust the language level in medical materials to make them more accessible to patients from diverse educational backgrounds [30]. Furthermore, AI has been used in developing patient education materials, as found in research on ostomy patients, where AI-generated materials were easier to understand and helped patients better comprehend their care [28]. This indicates that AI can play a crucial role in improving health literacy and assisting patients in making better decisions about their care.

### AI in Nursing Education: Enhancing Cognitive and Practical Skills

Generative AI technology and NLP-based chatbots are increasingly used in nursing education to enhance nursing students’ critical thinking, problem-solving, and communication skills. Studies show that ChatGPT can help students better understand nursing concepts and increase their engagement and enjoyment of learning [6].

Additionally, AI robots and chatbots have supported game-based learning and distance learning, which are becoming increasingly popular in medical and nursing education. Research trends show a rise in the use of AI in nursing education since 2018, with a primary focus on technology-based learning [19]. Meanwhile, AI-based virtual patients have also improved nursing students’ self-efficacy and confidence in communicating with patients. Nursing students practicing with AI systems demonstrated enhanced communication skills, which are crucial to their future clinical practice [22].

### AI in Clinical Decision-Making and Patient Monitoring

AI technologies based on ML, NLP, and robotic process automation have been instrumental in early disease detection, clinical decision-making, and patient monitoring. Studies show that AI can enhance diagnostic accuracy and expedite the decision-making process for nurses and doctors [8]. In neurology, GPT-4 in managing Alzheimer disease also demonstrates that AI can generate accurate medical information and assist in diagnosis and disease management. A comparison between AI responses and those from neurologists indicates that AI has the potential to become a valuable medical support tool in managing neurodegenerative diseases [29].

### Challenges and Prospects of AI in Nursing

Although AI holds significant potential in patient education within nursing care settings, several challenges must be addressed. Ethical issues, data privacy, information accuracy, and the limitations of AI in providing personalized approaches to patient education are significant concerns in its implementation. Some studies indicate that while AI can simplify medical information and enhance patient understanding, there are concerns that AI may diminish the humanistic aspect of communication between health care providers and patients [26]. Patient education relies not only on the information provided but also on emotional support and empathy, aspects that AI-based systems may not fully replace. Furthermore, another challenge is how AI can ensure the accuracy of the information provided, as large language model-based AI systems, such as ChatGPT and Google Bard, may sometimes generate invalid details or information that is less appropriate for clinical contexts [30].

Improve information accessibility, cognitive engagement, and perceived educational quality across diverse nursing contexts. However, outcome measurement remains inconsistent, with relatively few studies employing robust experimental designs or long-term follow-up. Most reported outcomes reflect short-term or context-specific benefits, highlighting the need for further research to evaluate sustained educational impact, behavioral change, and integration into routine nursing practice.

In teleconsultation, AI has helped improve communication efficiency between health care providers and patients, particularly in explaining medical conditions and treatment procedures. However, challenges remain in data security and legal responsibility, particularly regarding how the information provided by AI is managed and to what extent AI can be relied upon to deliver accurate medical education [25]. Additionally, in long-term care, research shows that AI is more widely accepted when used to monitor patient health rather than replace direct communication between health care providers and patients. This suggests that AI can enhance the efficiency of patient education systems. However, it must still be integrated with human interaction to ensure that patients fully understand medical information and feel emotionally supported during care [21].

## Discussion

### Principal Findings

This scoping review demonstrates that current applications of ChatGPT and related AI technologies in nursing are predominantly supportive in nature, functioning as adjunct educational tools rather than autonomous systems within clinical practice. Overall, the findings indicate that AI is predominantly positioned as a supportive educational and informational tool, rather than as an autonomous clinical decision-maker. This role alignment is consistent across diverse nursing contexts and reflects the early and exploratory stage of AI integration in health care education. Therefore, this review highlights 3 key aspects of AI application in nursing education: interpretation of AI use, the challenges encountered, and the implications of AI in nursing practice.

### Interpretation of AI Use in Nursing Education and Practice

Across diverse contexts, AI was primarily used to facilitate information access, educational clarification, and learner engagement, particularly under conditions of limited time and workforce capacity [7,25]. Rather than replacing professional judgment, AI tools are commonly described as supportive resources that improve efficiency and responsiveness, particularly in clinical and educational settings with limited time and high workload demands [20,33]. This pattern aligns with implementation science principles, which emphasize that digital innovations are most effective when embedded within existing professional workflows and governed by human oversight [17,18].

Similarly, ML-based systems and analytical tools have been applied to assist clinical monitoring, risk assessment, and decision support activities in nursing practice [21,27]. Importantly, the literature consistently emphasizes that these technologies are intended to support, rather than replace, nursing expertise. This reinforces the ongoing importance of professional responsibility and clinical judgment in patient education and care delivery, even as AI tools become more widely adopted [8,15].

### Challenges in the Implementation of AI in Nursing Education

Despite promising findings, the evidence base remains constrained by heterogeneity in study design and outcome measurement. Many studies relied on descriptive or qualitative approaches, limiting causal inference and generalizability. One of the main barriers is the lack of clear regulations and guidelines regarding the use of AI in health care services [20,34]. This lack of precise regulation leads to variations in the effectiveness of AI implementation across different health care and educational institutions. Some studies also indicate that AI has the potential to produce inaccurate or even misleading information, particularly in the context of telemedicine and AI-based consultations [25]. This presents a risk to patients who rely on AI as their primary source of medical information.

Additionally, ethical issues and data privacy are significant challenges in using AI in nursing education. This study reveals that the use of AI-based chatbots in mental health services continues to face challenges related to patient data security and the potential misuse of medical information provided by AI [35]. Therefore, measures are needed to ensure that the AI technology used in nursing education meets high standards of security and accuracy. From an implementation perspective, the findings suggest that AI adoption in nursing education should be accompanied by structured training, institutional guidelines, and continuous evaluation to ensure safe and effective integration into practice [26].

### Implications for Research and Practice

Overall, the findings of this scoping review suggest that AI-supported education has the potential to support nursing education and patient engagement, particularly as a complementary educational resource alongside nurse-led instruction. However, the available evidence remains limited and heterogeneous. Therefore, future research should prioritize implementation-focused studies, longitudinal designs, and standardized evaluation frameworks to assess the sustained educational and clinical impact of AI technologies in nursing practice.

### Recommendation

Based on the results of this scoping review, the following recommendations are proposed to guide future research, policy development, and implementation efforts in nursing education and practice.

#### Development of AI Regulation and Ethics Standards in Nursing

Further research should not only explore regulatory needs but also define the roles of key stakeholders in developing governance frameworks for AI use in nursing education and patient care. Regulatory development should involve professional nursing organizations, health care institutions, academic leaders, and health policymakers, who are responsible for establishing standards related to data security, information accuracy, accountability, and ethical use of AI-generated educational content.

#### Long-Term Evaluation of AI Effectiveness in Nursing Education

Instead of short-term outcome assessments, future research should prioritize longitudinal and implementation-oriented study designs to evaluate the sustained educational impact of AI-supported learning. Researchers, nursing educators, and health care organizations should collaboratively assess how AI tools are integrated into curricula, clinical training, and continuing professional development, including evaluation of learner outcomes, professional competence, and unintended consequences over time.

#### Optimization of AI for Personalized Patient Education

The optimization of AI for personalized patient education should move beyond technical development alone and be guided by clinical oversight and educational design principles. AI developers, nurses, and patient education specialists should work together to design adaptive learning models that tailor information based on patients’ literacy levels, clinical conditions, and cultural contexts, while ensuring content validation by HCPs. Implementation strategies should include pilot testing in clinical settings, training programs for nurses as end users, and mechanisms for continuous monitoring and feedback.

### Limitations

This scoping review has several limitations that should be considered when interpreting the findings. While challenges related to AI implementation, such as ethical concerns, data privacy, and the potential generation of inaccurate information, are frequently discussed in the included literature, these issues were not empirically evaluated within this review and therefore could not be assessed for their direct impact on educational effectiveness.

In addition, the findings of this scoping review are influenced by limitations in the search strategy and inclusion criteria. The literature search was restricted to studies published between 2019 and 2025, written in English, and available in full text, which may have resulted in the exclusion of relevant studies published in other languages or earlier periods.

Furthermore, only 3 electronic databases were searched, which may have limited the comprehensiveness of the evidence captured. Although this approach aligns with scoping review methodology, it may have led to the omission of relevant gray literature or discipline-specific publications.

The included studies also demonstrated substantial heterogeneity in study design, context, AI technologies, and outcome measures, which limited the ability to compare findings across studies or draw conclusions regarding effectiveness. As a result, the synthesis was descriptive rather than evaluative, consistent with the exploratory purpose of a scoping review.

### Comparison With Prior Work

The scoping review compares its findings with prior work, noting that while AI, particularly ChatGPT, shows significant promise in nursing education, the technology faces challenges similar to those outlined in earlier studies. Previous research also points to concerns about AI’s capacity to replace human interaction in patient education. The review emphasizes that AI’s effectiveness in improving clinical decision-making and patient monitoring has been widely acknowledged, but further development and regulation are necessary to address the ethical and accuracy concerns highlighted by earlier work.

### Conclusions

This scoping review mapped the emerging evidence on the use of ChatGPT as an educational resource in nursing practice and education. The findings indicate that ChatGPT is primarily applied as a supportive, adjunct tool to facilitate information access, educational clarification, and learner engagement, rather than as an autonomous or decision-making system.

However, the current evidence base is heterogeneous and largely exploratory, with limited empirical evaluation of long-term educational or clinical outcomes. Ethical considerations, data privacy, and the need for professional oversight remain critical challenges for implementation.

Future research should prioritize implementation-oriented and longitudinal studies to clarify how ChatGPT and similar AI technologies can be responsibly integrated into nursing education and practice. In particular, standardized outcome measures, clear governance frameworks, and evaluation of professional oversight mechanisms are needed to assess sustained educational value and contextual appropriateness across diverse nursing settings.

## Supplementary material

10.2196/79551 Multimedia Appendix 1 PRISMA-ScR checklist.

## Abbreviation

AI: artificial intelligence
HCP: health care professional
ML: machine learning
NLP: natural language processing
PRISMA-ScR: Preferred Reporting Items for Systematic reviews and Meta-Analyses extension for Scoping Reviews
